# Adolescent idiopathic scoliosis – to operate or not? A debate article

**DOI:** 10.1186/1754-9493-2-25

**Published:** 2008-09-30

**Authors:** Hans-Rudolf Weiss, Shay Bess, Man Sang Wong, Vikas Patel, Deborah Goodall, Evalina Burger

**Affiliations:** 1 Asklepios Katharina Schroth Spinal Deformities Rehabilitation Center, D-55566 Bad Sobernheim, Germany; 2 Colorado Spine and Scoliosis Institute, Lonetree, CO, 80124, USA; 3 Department of Health Technology and Informatics, The Hong Kong Polytechnic University, Hong Kong, PR China; 4 Departmet of Orthopedics, University of Colorado School of Medicine, Denver, CO, USA; 5 Rehabilitation services, Ealing Hospital, Uxbridge Road, Southall, London, UB1 3HW, UK

## Abstract

Adolescent idiopathic scoliosis (AIS) represents a rare condition with a potentially detrimental impact on young patients. Despite vast clinical research and published treatment guidelines and algorithms, the optimal therapeutic choice for these patients remains highly controversial. While advocates of early surgery emphasize the benefits of surgical deformity correction with regard to physical and psychological outcome, the opponents base their arguments on the high risk of complications and a lack of documented subjective long-term outcome. In the present paper, the authors were invited to debate the opposite positions of "pro" versus "contra" surgical treatment of AIS, based on the currently available evidence and published guidelines.

## Introduction

Adolescent idiopathic scoliosis (AIS) is a three-dimensional spinal deformity with potential adverse long-term physical and psychological impact on young patients. Surgical treatment of spinal deformity remains an emotional decision for both the patients and their parents. Positive results from non-operative treatment modalities have been increasingly reported in the peer-reviewed literature. Thus, the decision-making process for operative vs. non-operative treatment should be based on the individual patient's disease characteristics, curve type, risk factors for progression, and subjective expectations. In the present paper, two groups of experts in the field for operative and non-operative treatment of AIS were invited to debate this important topic from an evidence-based perspective. The aim of this debate article is to provide a balance between the available treatment options and help as a guide in the decision making process for this uncommon, but potentially disabling disorder in young patients.

## Debate: "Pro" surgery

**Figure 1:**
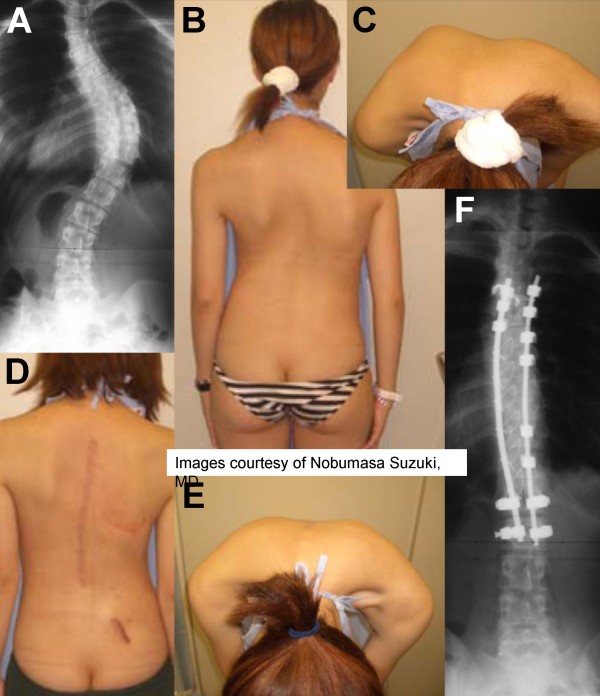
**Case example of a 19 year-old old female with a curvature of 56 degrees (preoperative panels, A-C).** A surgical deformity correction was performed. At 6-months follow-up, the patient has a perfect correction of the curve cosmesis and rib hump (postoperative panels, D-F). The pictures were kindly provided by courtesy of Dr. Nobumasa Suzuki.

**Figure 2:**
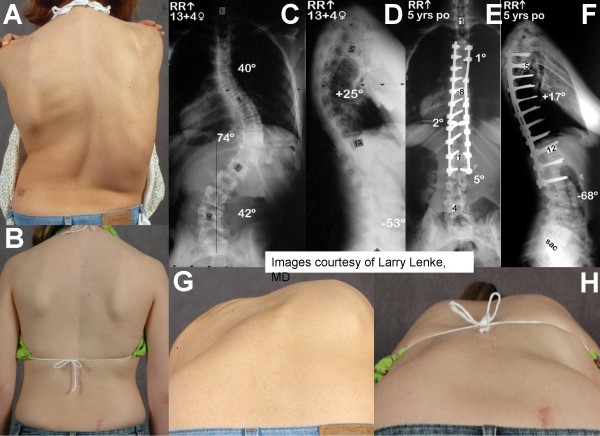
**Case example of a 13 year-old old female with progressive AIS.** Clinical photographs show spinal imbalance, and elevated right shoulder (preoperative panels A, G). Adam's forward bend test shows right thoracic prominence correlating with vertebral rotation and right rib elevation. Radiographs in postero-anterior and lateral views show a proximal thoracic curve of 40°, a main thoracic curve of 74°, and a lumbar curve of 42° (panel C), with normal sagittal parameters (panel D). A posterior correction with fusion from T4-L2 was performed. Five years postoperatively, radiographs show a 97% correction of the proximal and main thoracic curves with a good maintenance of sagittal balance (panels E, F). Clinical photographs at 5 years show a restoration of spinal balance (panel B). The Adam's forward bend shows reduction of thoracic rib prominence (panel H). The pictures were kindly provided by courtesy of Dr. Larry Lenke.

**Figure 3:**
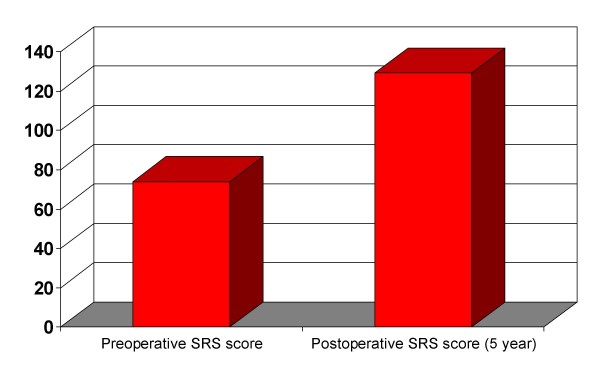
Preoperative and 5-year postoperative Scoliosis Research Society (SRS) score of the patient shown in figure 2, showing a subjectively improved health-related quality of life (HRQL).

In order to promote an intervention for a specific condition, it must be demonstrated that; 1) the natural history of the condition is undesirable, 2) the intervention alters this natural history in a favorable and reproducible manner, 3) the complications are minimal, and 4) the long term side-effects of the intervention are not detrimental, so that the risk-benefit ratio favors the intervention over the condition's natural history. Scoliosis is a general term indicating a lateral curvature of the spine due to a variety of etiologies. Each etiology has a specific natural history and, consequently, has its own unique treatment goals, and risk-benefit and treatment efficacy profile. Early studies that investigated the natural history of untreated scoliosis and complications associated with treatment, contain study populations of mixed scoliosis etiology, and, therefore, have provided somewhat tainted conclusions clouded by heterogeneous study populations [1-4]. The purpose of this argument is to demonstrate that 1) spinal deformity associated with AIS can increase, 2) specific risk factors have been identified that reliably predict curves at risk for progression, 3) the natural history of untreated, progressive scoliosis is unfavorable and, 4) surgical treatment of progressive AIS is safe, consistently achieves the goals of treatment, and favorably alters an otherwise negative natural history in a measurable fashion (figures 1, 2, 3).

### Identification of patients at risk of progressive spinal deformity

The Scoliosis Research Society (SRS) definition of AIS is a structural lateral curvature of the spine that creates a thoracic and/or lumbar asymmetry on forward bending (a.k.a. the "Adams forward bend test") combined with a curve of at least 10° as measured by the Cobb technique on a standing radiograph of the spine with associated vertebral rotation. The diagnosis of the curvature must occur after the age of 10 years, and there is no other established etiology for the curve [5]. Given this definition, it has been repeatedly demonstrated that AIS curves can and will progress. Curve progression is defined as an increase in curve magnitude of 5–10° on serial radiographs. The primary factors for AIS curve progression include growth potential and curve magnitude.

#### Growth potential

Duval-Beaupere initially reported the correlation between skeletal growth and curve progression, indicating that curve progression occurs most rapidly during the adolescent growth spurt. However this study was confounded by inclusion of post-polio and AIS patients in the study population [6]. Subsequent reports that focused exclusively upon AIS and curve progression have demonstrated that curve progression is dependent upon the child's remaining growth, and have identified specific radiographic and clinical indicators for skeletal maturity and the risk for curve progression [7,8]. Lonstein and Carlson used the iliac apophysis ossification (Risser sign) and onset of menses as indicators for skeletal maturity to correlate curve magnitude and skeletal maturity with curve progression in 727 patients with AIS [8,9]. Immature children (Risser sign 0 or 1) with larger curves (20–29°) at initial diagnosis demonstrated a 68% risk for curve progression, whereas mature children (Risser 2–4) with similar curves at initial presentation had a 23% risk for curve progression. Conversely, immature children with smaller curves (5–19°) demonstrated 22% chance for curve progression, while mature children with smaller curves had only a 1.6% risk for curve progression. Sixty-eight percent of girls with non-progressive curves had onset of menses prior to the initial assessment. More recently, Sanders and coworkers contributed substantially toward identifying the most valid and reproducible indicators of skeletal maturity and correlating skeletal growth with curve progression [10]. The authors evaluated different radiographic measures, secondary sexual characteristics, and serum markers to identify the most accurate predictor of skeletal maturity and the relationship of these markers to the child's peak height velocity (PHV; the maximal adolescent growth height velocity) [10]. The Tanner Whitehouse III RUS scoring system (which uses the radiographic appearance of the epiphyses of the hand, distal radius, and distal ulna to generate a skeletal maturity scoring system) was the most predictive and reliable method to determine the patient's chronological relationship to PHV. Subsequently, the authors evaluated scoliotic curve behavior and reported that progressive curves demonstrated consistent behavior with reproducible periods of progression, the most rapid period of progression was termed the curve acceleration phase (CAP) [11]. Different criteria were then evaluated to predict the relationship of the child's remaining growth to the CAP. Again, the Tanner Whitehouse III RUS scoring system correlated most accurately with the the child's maturity and the CAP. The authors developed a simplified version of Tanner Whitehouse III RUS scoring system, and were able to correlate the appearance of the hand epiphyses with different stages of skeletal growth and also these growth stages with the CAP [11]. Sanders *et al. *have since developed a predictive model that correlates hand epiphysis scores with scoliosis progression, and is able to pair the stages of skeletal maturity with the probability of scoliosis progression beyond 50° [12]. In their study, patients at greatest risk for curve progression beyond 50° were children that presented with curves > 20° and were hand stage 2, and children that were hand stage 3 with curves > 30°.

#### Curve magnitude

The size of a scoliotic curve is an independent predictor of curve progression. Nachemson et al, and Weinstein et al, correlated curve progression with age and curve magnitude, demonstrating the importance of age and curve magnitude when predicting curve progression [13-16]. However, it has been demonstrated that curve magnitude is an independent predictor of curve progression and that larger curves can progress after skeletal maturity. Weinstein *et al. *and Ascani and colleagues reported that children with curves < 30° at skeletal maturity did not demonstrate curve progression into adulthood, while the majority of curves > 50° progressed at approximately 1° per year [15-17]. Consequently, patient counseling usually involves discussions indicating that curves that are < 30° at skeletal maturity are at low risk for progression, while curves > 50° are at risk for progression into adulthood [16,18].

### Impact of progressive spinal deformity on the quality oflife

The question of untreated AIS causing pain, disability, and negative impact on quality of life in adulthood has demonstrated conflicting results. Early studies reported that untreated scoliosis resulted in increased back pain and disability, negative socioeconomic effects on work and marriage, and early mortality compared to controls [3,4,19,20]. As previously indicated, however, these studies had mixed etiology of scoliosis, including infantile and juvenile idiopathic scoliosis. Recent work by Weinstein and coworkers re-evaluated the amount of disability that adults with untreated AIS incur. Fifty year follow up of 117 patients with untreated AIS (average curve magnitude > 75° for thoracic, thoracolumbar, and double major curve patterns) demonstrated no increase in mortality rates, disabling back pain, and ability to complete daily activities compared to age and gender matched controls [18]. However, acute and chronic back pain, and dissatisfaction with appearance were more prevalent in patients with scoliosis, as was shortness of breath in patients with thoracic curves > 80°. These findings demonstrate that the prognosis for untreated AIS is not as poor as was originally reported, however their findings further demonstrate that patients with untreated AIS are unquestionably dissimilar to matched controls. The authors acknowledge that the clinical significance of their report is to differentiate the natural history of AIS from the more negative prognosis for untreated infantile and juvenile idiopathic scoliosis patients, rather than demonstrate equality between patients with and without AIS. A growing body of literature does indicate that adults with scoliosis demonstrate greater function limitation, greater daily analgesic use, and less satisfaction with their appearance compared to unaffected individuals [19,21-23]. In an attempt to quantify the amount of disability associated with scoliosis, Berven et al used established "health-related quality of life" (HRQL) outcomes measures, including the SRS research instrument (SRS-22), to evaluate pain and function in adult patients with and without scoliosis (figure 3). The SRS research instrument was originally developed to evaluate surgical outcomes for AIS in children [24-26]. This instrument has subsequently been validated as the standard measure for AIS surgical outcomes and disability associated due to AIS, with validated translations in Chinese, Japanese, Spanish, and Turkish. The SRS questionnaire has also demonstrated differences in function, pain, and self-image between children with and without scoliosis, as children with AIS demonstrate lower scores than controls in all domains across cultures [26-35]. In an attempt to extrapolate these data to adults, Berven and colleagues demonstrated that the SRS-22 is a reliable instrument for measuring disability associated with adult spinal deformity, reporting SRS-22 criteria to be valid in use with the SF-36 [21]. The authors reported that adults with scoliosis scored significantly worse on every SRS-22 and SF-36 domain (including pain, function, self-assessment, and mental health), compared to controls with no scoliosis. These findings have subsequently been supported by Bridwell *et al.*, validating the SRS-22 as an accurate measure of function and disability in adults with scoliosis, and that the SRS-22 demonstrates greater accuracy and consistency for measuring outcomes for the treatment of adult scoliosis compared to previous HRQL measures including SF-12, SF-36, and Oswestry Disability Index (ODI) [29].

### Surgery for AIS is safe and efficient

The primary surgical goals for treating AIS are to prevent curve progression, obtain solid arthrodesis, and restore spinal balance. A plethora of literature has repeatedly demonstrated that, if established surgical guidelines are followed, surgical arthrodesis prevents AIS curve progression, restores spinal balance, and, when permissible, preserves spinal motion segments via selective fusion of structural curves and indirect deformity correction of compensatory curves [36-38]. Using modern surgical techniques (i.e. segmental spinal fixation, meticulous posterior element decortication, and bone grafting) solid arthrodesis is achieved in over 95% of AIS cases [39,40]. Additional goals addressing comesis and self-image concerns are also addressed, as current surgical techniques and have demonstrated improved curve correction and cosmetic outcome compared to early surgical techniques [40-43].

Reported perioperative complication rates following surgery for AIS range between 5% and 20%, depending upon the surgical approach, and differentiation between major and minor complications [40,42,44,45]. The most recent SRS morbidity and mortality committee update reported a 5.7% surgical complication rate in 6334 patients treated for AIS from 2001–2003 [45]. Recorded complications included pulmonary, infection, neurological deficit, dural tear, deep venous thrombosis, and death, but were not divided into major and minor complications. The incidence of complications following posterior spinal fusion was 5.1% whereas the incidence following combined anteroposterior spinal fusion was 10.2%. Wound complications were the most common complications for PSF (1.4%), and pulmonary problems were the most common for anterior procedures (1.6%). Neurologic complication rates for anterior, posterior, and combined procedures were 0.26%, 0.32%, and 1.75%, respectively. Diab et al, reported 0.69% rate of neurological complications in 1,301 surgically treated cases of AIS [46]. These findings were corroborated by a 1.06% rate of neurological complications following surgery for AIS at a Chinese hospital over a 7 year period [47]. Carreon and colleagues prospectively evaluated the prevalence of non-neurologic complications following surgical treatment of AIS, reporting a 15.4% prevalence in 702 patients treated from 2002–2004 [44]. The reported prevalence of major complications including major blood vessel injury, visceral injury and deep infection was 0%, 0.28% and 0.71%, respectively. The majority of compilations (3.7% prevalence) were labeled "miscellaneous", including transverse process failures, ileus, lateral femoral cutaneous nerve dysesthesia, diarrhea, and allergic reactions to medication. There was no significant association between the prevalence of a non-neurologic complications and curve pattern, curve magnitude, surgical approach, number of spinal levels fused, and type of bone graft used.

Autograft harvest site pain is often reported as a common complication and source of morbidity following spinal arthrodesis, however a separate bone graft harvest might not be necessary when treating AIS. Betz *et al. *reported equivalent arthrodesis rates for allograft vs. no graft in a prospective, randomized study that evaluated grafting techniques and fusion rates following posterior spinal fusion for AIS [39]. The authors reported a 1.3% pseudarthrosis rate, minimum 2 year follow up, among 76 AIS patients. Patients randomized to no graft demonstrated no pseudarthrosis compared to one patient randomized with allograft, who developed a non-union.

Finally, in order to advocate for surgical treatment of AIS, it must be demonstrated that the consequences of surgical treatment in adolescence are not detrimental in adulthood. Long term studies from Canada and Sweden have indicated that adult patients treated with spinal fusion as adolescents report reduced function and greater frequency of back pain compared to controls [48,49]. However, the reported severity of reported back pain was mild and daily back pain was uncommon. Additionally, it is difficult to ascertain if these limitations were due to surgical treatment of scoliosis or due to scoliosis itself, as it has been reported that patients treated surgically for AIS indicate greater function and less pain than patients treated non-operatively, however function and pain levels continue to remain inferior to controls without scoliosis [22]. As indicated above, adolescents treated surgically for AIS demonstrate significant improvement in HRQL measures compared to preoperative scores, including improvement in all SRS questionnaire preoperative domains at 2 year follow-up, and report high treatment satisfaction levels [26]. Of note, Upasani et al reported that AIS patients demonstrated significantly increased back pain 5 at year follow-up compared to 2 postoperative values [50]. The etiology of this increase in pain was not determined, nor was the clinical significance of the increase in pain, namely if it reached a so-called "minimally clinical important difference". Even so, pain scores at 5 year follow up were still lower than preoperative values, and patients continued to reported high treatment satisfaction and high self-image, independent of the pain scores.

### Discussion

Surgical treatment of spinal deformity remains and emotional decision for both the patients and their parents. In our modern society, the affected child's parents have to assume the responsibility for making a decision that could influence their child's life forever. It is therefore necessary to define the roles for each treatment option and to define the expected long-term outcomes for each treatment modality. Many adults are now presenting for scoliosis surgery who still remember their childhood years spent in casts and turnbuckle braces. The advancement of general medicines are keeping these patients alive and they are in chronic pain and disabled. They have been able to live a relatively productive life and are now faced with the late effects of untreated scoliosis, quite bitter that they were denied the surgical option by their parents when they were young. It is hard to balance this argument when faced with a young teenager as it is impossible for us to see what future development might bring in term of revolutionizing the disease. It is agreed from both view points that the disease warrants treatment. We therefore suggest that surgical and non surgical treatment option each has their rightful place and that the decision for treatment should be based on the curve type and the risk factories for progression, keeping in mind the end result as well as the expected long-term outcomes (figures 2, 3).

## Debate: "Contra" surgery

### Signs and symptoms of scoliosis cannot be changed by spinal fusion surgery

Spinal fusion surgery, which is recommended when the magnitude of curvature exceeds 40–45 degrees, has been used as a treatment for nearly a century [51-53]. The aims and goals of surgery have varied widely. The early belief was that spinal fusion could be used to leave the patient with a mild residual deformity but this is not the case as one third of patients lost all postoperative correction within one to ten years post surgery [54]. Expectations have been revised to the more modest goals of preventing progression, restoring 'acceptability,' and reducing curvature. In spinal fusion, the vertebrae are accessed by posterior, anterior, or thoracoscopic incision. The main principle of these surgical techniques is to use the spine as a structural scaffold, cementing the parts onto this via a bone paste, giving it an overall straighter shape. [53,55-57]. These surgical methods are based on the assumption that this will heal well and remain sturdy for patient's lifespan. Metal rods, screws, wires etc. have been used to reinforce the stability of the spinal fusion [56-61] and the choice of instrumentation is based upon the preferences of the surgeon [62-66]. Failure of spinal fusion requires re-operation to restore curvature correction [62].

What specific evidence is there to support scoliosis surgery? The signs and symptoms of scoliosis obviously cannot be changed by scoliosis surgery and long-term beneficial effects have not been reported yet [67,68] and, in addition to this, there are no studies presenting the long-term risks [67]. Moreover, no report of the long-term surgical outcome (balance, rate of fusion, rib hump correction, cosmetic correction, pain, and patient satisfaction) is available for any study series. Further prospective studies including these parameters will be required to determine the real benefit of such procedures for the patient [67].

### No evidence for surgery in prospective controlled trials

There are in fact prospective controlled studies comparing the outcome of patients with AIS treated conservatively with a series of patients treated surgically [48,69-73]. Nevertheless no study is available comparing surgery to the natural history prospectively [74-76]. The Gothenburg's papers do not offer any evidence that the long-term outcome of surgery is superior to the long-term outcome of patients treated conservatively [48,69-73]. The studies relating to HRQL/SRS-22 questionnaires do not demonstrate differences between the two groups of patients [70], pain and function do not differ [48,73], nor does degeneration [71], sexual function [72] or restrictive ventilation disorder [69]. As early as 1973, Paul Harrington envisioned in the future a common database or registry of all Scoliosis Research Society (SRS) members' patient's treatment results [51]. Unfortunately the SRS failed to follow this vision until recently. Instead of achieving long-term evidence for surgical treatment on a higher level and addressing the problems after surgery to attempt to improve patient safety, the surgical community is presenting large numbers of papers describing HRQL after surgery and related research [26,77-81].

The problem with such studies is that they lack validity as they do not investigate the actual signs of scoliosis or the post-surgery symptoms of the patient [82]. Those studies containing psychological questionnaires may be compromised by the dissonance effect [74-76,82,83]. This implies that all situations which include important decisions to be made. Cognitive dissonance occurs most often in the situations where an individual must choose between two incompatible beliefs or actions and there is a tendency for individuals to seek consistency among their cognitions. Unable to face an inconsistency, such as being dissatisfied with a surgical procedure, an individual will often change an attitude or action. Surgery is impossible to reverse, but subjective beliefs and public attitude can be altered more easily. The clinical significance of this is that a patient dissatisfied with surgical treatment may not necessarily admit this publicly, as the findings of the following studies:

*"Slim objective favorable outcomes correlate with high post-surgical patient satisfaction, while a considerable share of patients with whom a highly favorable outcome has been attained express relatively low post-surgical patient satisfaction. This paradoxical trend may be well understood when applying Cognitive Dissonance Theory. The whole pattern of results point again at highly complex and powerful psychological processes, some of them seemingly irrational" *[82].

*"Patient satisfaction is subjective. It does not reflect the benefits of surgery with respect to the future preservation of pulmonary function in thoracic curves nor the prevention of osteoarthritis in lumbar curves" *[84].

*"Radiographic and physical measures of deformity do not correlate well with patients' and parents' perceptions of appearance. Patients and parents do not strongly agree on the cosmetic outcome of AIS surgery" *[85].

From searching all of the studies based on questionnaires within this review, no evidence can be derived that supports the assumption that patients have experienced benefits from undergoing surgery, as none were able to rule out the cognitive effect of dissonance. Without being able to rule out such effects on the post-operative experience these outcomes do not appear to be valid for the group of scoliosis patients treated surgically [74-76].

### Complications of spinal fusion surgery

In principal, all kinds of complications may occur in all scoliosis aetiologies [67]. However, in the otherwise healthy subjects with AIS the incidence of major complications may not be as high as in neuromuscular disorders [67,86]. Risks of spinal fusion include those occurring in any major surgery such as severe blood loss; urinary infections due to catheterization; pancreatitis; and obstructive bowel dysfunction due to immobilisation during and after surgery [67,86]. The frequency of specific complications, including death is unknown. This is due to problems in reporting such as; mandatory reporting, definitions, interpretation of complications and variations within compliance [67]. Information is based on voluntary reporting by clinicians. Other risks of scoliosis surgery, as listed in recent reviews [67,86] are summarised below:

#### Death and neurological damage

The incidence of death as a complication of spine surgery, for otherwise healthy patients is reported to be less than 1%. In one survey only one child out of 352 patients died of peritonitis and in a group of 447 patients, 2 deaths occurred due to pulmonary complications. The life expectancy of patients with complex neuromuscular condition was significantly reduced by spinal surgery. Another study involving adults with a less than 60% vital capacity measure, 20% had died within 1 year post surgery. In a survey further highlighting these complications, 21% were contributed to be secondary to spinal fusion surgery [86].

Symptoms of neurological damage post-surgery include; partial or total paraplegia, quadriplegia, or peripheral nerve deficit. Neurological deficits can result from vascular, metabolic, or mechanical complications of spine surgery. Published cases include migration of bone graft into the spinal canal; breakage of implants; penetration of instrumentation into the spinal canal and compression of the nerve roots by components of implants [86].

#### Loss of normal spinal function

In every case of spinal surgery there is an irreversible loss of the normal active range of movement in the spinal column, including the non-fused segments. When compared with control subjects, the ability of surgical patients to side flex was reduced by 20–60%. This loss of spinal mobility has gained little attention in the literature especially in relation to the detrimental effects upon patient's health, function, and quality of life. Winter and colleagues [87] argued that *'it has long been a clinical observation by surgeons who manage scoliosis that patients seem to function well and be relatively unaware of spinal stiffness, even after many motion segments have been fused.' *No data in support of this observation has been provided [67]. In fact, it has been shown that pain increases as flexibility is reduced in non-surgical cases [88].

#### Strain on adjacent, non-fused vertebrae

The post-surgical rigid spine causes strain on the un-fused parts of the skeletal framework. More commonly reported are post surgical degenerative changes, which occur in young adults and older adults, sometimes within 2 years post-surgery. A higher degree of correction results in a higher rate of degenerative osteoarthritis, and the high stress on the rigid spine can cause serious injuries [86].

#### Post-surgical pain

Pain is the primary indication for re-operation [67,86]. The mechanism for increased neck and back pain after surgery is not well understood. Bridwell [53] suggests that late-developing pain could be a complication of surgery, or an effect of aging, or *'perhaps a focus on the disability associated with spinal deformity and surgical treatment'*. Among 190 patients, 19% required re-operation within 2 to 8 years after surgery. For 27 patients who sought treatment 59% felt their pain had been reduced, but 41% did not feel a reduction in their pain levels, and a further 26% were very unhappy with the outcome [86]. Among 34 patients with significant post surgical pain, 56% reported reduced pain after additional surgery, while 44% did not; within the same study, 2 patients who did not have pain before surgery reported pain in follow-up [86]. Pain at the iliac graft site, first noted in 1979, has now been formally published; of 87 patients, 24% complained of pain at the graft site, with 15% reporting severity sufficient to interfere with daily activities. As reported by the authors such problems with iliac crest grafting have been severely neglected in literature, especially problems associated with rib-resection [67,86].

#### Infection and inflammatory processes

Infections from surgery may manifest months or years later and has been detected more than 8 years after surgery, with 5 to10% of patients developing deep infections at 11–45 months after surgery and in some cases leave the spinal cord exposed to injury. Infections become more common, perhaps due to larger instrumentation used or perhaps due to the increasing prevalence of multi-drug resistant bacteria in hospital settings. Inflammatory responses to metallic instrumentation can occur independently or in conjunction with infections. In most cases, additional surgery to remove instrumentation and to treat the wound is required [67,86]. Infection also may be transmitted through blood transfusions needed to replace the large amounts of blood lost during invasive procedures and a similar risk occurs with the use of allograft. Some have reported to be infected with HIV following this type of surgery. In a survey of spine surgeons, 41% of those using allograft reported having concerns about the risk of disease transmission and 88% of those make it a policy to inform parents [67,86].

#### Curvature progression

Some curvatures continue to progress after spinal fusion due to broken rods or other failure of instrumentation. Renshaw [56] has said that, *"One would expect that if the patient lives long enough, rod breakage will be a virtual certainty." *Furthermore, discomfort may occur when any pressure is placed against the back; this is especially problematical with newer bulky instrumentation implanted in thin patients [53]. Pseudarthrosis or failure of the bone graft, which constitutes the spinal fusion, can occur years after surgery and can be difficult to diagnose. Among 74 patients treated surgically between 1961 and 1976, pseudarthrosis occurred in 27% of patients within a few years of surgery. For adult patients, 15% had failure of fusion and/or instrumentation requiring additional surgery. Curvatures may continue to progress in young children despite a rigid fusion, due to a *'crankshaft phenomenon' *in which spinal growth causes rotation around the fusion [67,86].

#### Decompensation and increased sagittal deformity

Beginning with Harrington's rods, surgeons have experimented with instrumentation of increasing complexity and bulk to hold spinal fusions in place. Each new instrumentation has brought with it new problems. One of the ongoing problems is decompensation or the development of new deformities involving changes in sagittal contours and coronal balance of the body as a result of surgery. Reducing the lateral curvature in thoracic scoliosis can exacerbate the sagittal deformity and cause flattening of the cervical, thoracic and or lumbar spine beyond that cause the deformity itself. Development of *'flat back' *is a painful condition with potentially devastating complications such as disability. In response to such discoveries, focus is shifting towards the sagittal contours and coronal balance of the spine [53,67,86].

#### Increased torso deformity

**Figure 4:**
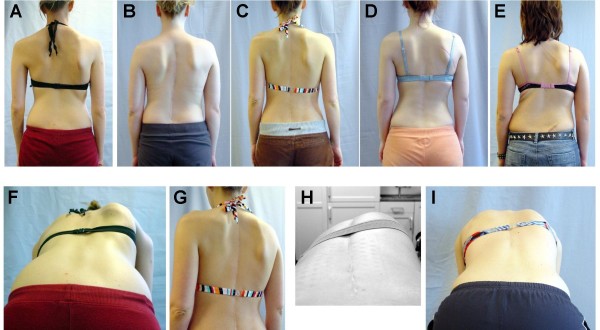
**Selected case examples of patients who underwent spinal fusion surgery for curvatures between 50 and 60 degrees (upper panels, A-D).** As shown in the examples in the lower panels (F-I), a ribhump may remain visible despite surgical correction. The patient in panel E was operated for a curve of 32 degrees thoracic and 28 degrees lumbar and had a progression of the thoracic curve to more than 50 degrees after operation. As all of the patients on this picture are still young, a further clinical (and radiological) deterioration may be expected.

Despite the application of force to straighten and de-rotate the spine during surgery, the rib hump can deteriorate after surgery. Even when rib hump magnitude improves postoperatively, much of the correction can be lost and in many patients appears worse than before the surgery (figure 4, panels A-E). In response, surgeons increasingly use costoplasty to assure an improved appearance, by excising the ribs that comprise the prominence. This procedure can in actual fact cause a progressive scoliosis [52] and the destabilising effects of rib removal can also result in a disabling condition called *'flail chest' *in which the normal function of the rib cage is permanently compromised. Rib resection excises a substantial part of the functional components of the chest but the effects on chest expansion have not been documented. However, this procedure has been shown to reduce the volume of the chest cage and to substantially impair pulmonary function [67,86].

#### Other long-term complications

The complexity of spinal surgery is reflected in the diversity of complications that may result months or years later. Given the time delay and difficulty in diagnosis, it is likely that only a minority of such events are recognised as surgical complications and when investigated are then recognised as being related to the surgery [67,86].

#### Salvage surgery

Due to such complications outlined above more re-operation is necessary, sometimes referred to as 'reconstructive', 're-corrective,' 'revision,' or 'salvage' surgery. Even stable fusions may fail in response to sudden force, as in automobile accidents. Some authors suggest that patients and their parents should be advised that it may take more than one operation [67]. Documented cases of having 5 or more salvage surgeries, as in one study, 22% of patients needed a total of 28 additional operations and of 110 adolescent patients, 21% required implant removal [89]. Complication rates vary; failure of fusion has been found in more than 50% of treated patients [67] and among 25 adult patients, 40% required salvage surgery. Even when a solid fusion has been obtained by the time of re-operation, removal of instrumentation *'may lead to spinal collapse and further surgery' *[67,86].

### Discussion

From the patient's perspective, the preferred plan of action would likely be based upon avoiding unnecessary risk i.e. avoiding surgery, or to keep it as the final option, once all conservative measures have failed. Under this premise, every effort should be undertaken to improve non-operative treatments for AIS, the most common form of scoliosis, which is regarded to be relatively benign [18]. In view of the fact that there is no evidence that health related signs and symptoms of a scoliosis can be changed by spinal fusion in the long-term [67,74-76], a clear medical indication cannot be derived from most scoliosis conditions [74-76]. In the light of an actual publication on adolescent idiopathic scoliosis with a prospective design [44], showing the short-term risks of scoliosis surgery to be more than 3 times higher than previously expected from retrospective reviews, the matter of surgical indications at present should be investigated more closely in order to improve the safety for patients, however, as has been shown [86], still today there are more open questions, than answers regarding the outcome of spinal fusion surgery for AIS patients.

**Figure 5:**
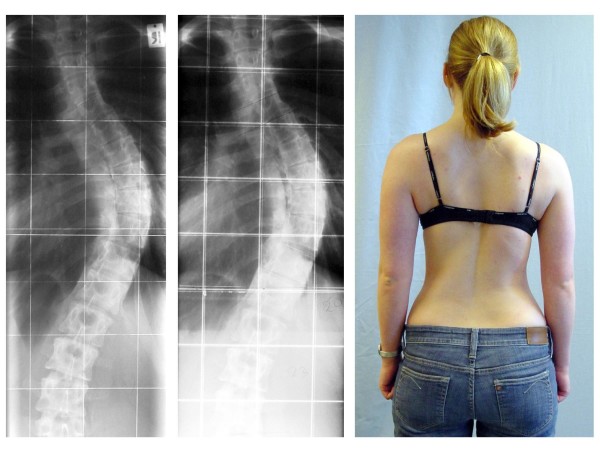
A young female patient with initially 56 degrees thoracic curve at the age of 15 years, treated with a Chêneau brace and scoliosis intensive rehabilitation (SIR) at the age of 20 (right panel), currently with 47 degrees and a clinical appearance comparable to the postoperative appearance shown in figure 4.

**Figure 6:**
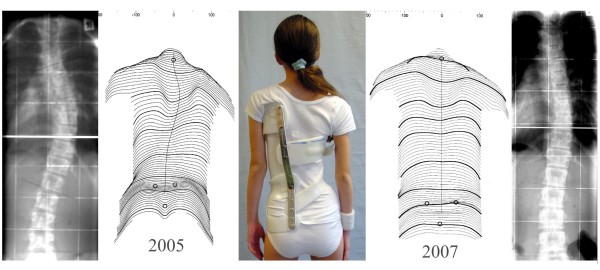
**Case example of an 11 year-old girl with 38 degrees curvature, Tanner II, corrected to 19 degrees after two years of brace treatment (2005–2007), followed by part-time bracing.** Clinically, the patient has improved significantly. Current braces work with much less material than conventional braces, thus providing more comfort to the patients.

In consideration of the questions generated by research [67,74-76,86], the lack of measurable medical benefit [67,90] and the high amount of short and long-term risks of the surgical procedures applied, the decision to have surgery does not rely on any valid evidence to support it. The informed patient perhaps should make the final decision after being provided with all the objective facts available. There is evidence on physical therapy to be found [91] justifying a grade A recommendation while conservative management [75] to be a grade B recommendation and spinal fusion surgery is considered as only a grade C recommendation [67,74-76,90]. Some spine surgeons still do not acknowledge the role of conservative management although evidence exists [92], reasons for this are perhaps based in an underlying conflict [93]. Today's "best practice" bracing technology enables to provide significant clinical and radiological improvements (figures 5, 6) [94,95], which clinically are comparable to what is achievable by applying spinal fusion surgery (figure 5, compared to figure 4). The current development of scoliosis braces seems to have a less negative impact on quality of life than braces of previous standards [96] using less physically restrictive material but meeting the same in-brace correction effects (Figure 6) [95]. Physical therapy due to present evidence has to be regarded to be effective in preventing curvature progression in smaller curves [75,91]. Therefore, spinal fusion surgery seems not to be indicated medically, while in bigger curvatures the patients themselves should understand the complications and decide whether their deformity is having too much of a negative psychological impact to resort to such invasive treatment. Spinal fusion surgery for AIS patients should be performed only after describing all the adverse effects the operation might have [67,86] to enable the patient to weigh the risks against the benefits in the long term [67]. Conservative management involves very different skills from surgery, which may sway surgical opinions towards a negative perspective on this subject [92]. However, anyone claiming to be a specialist in the treatment of scoliosis should advise patients according to evidence based practice and should offer a high quality conservative management to the patients.

## Conclusion

### "Pro" surgery

AIS is a relatively uncommon condition, occurring in approximately 2–3% of children. The number of children that require treatment for AIS, is even smaller, approximately 0.1–0.3%, and those that receive surgical treatment is smaller still^27^. However, progressive AIS is not a benign condition and should not be observed. Children with progressive AIS report lower self-image and worse HRQL scores than controls with no scoliosis. These low scores translate into further disability and dissatisfaction into adulthood. Although recent studies have indicated that the prognosis for untreated AIS in adults is not as dire as was originally thought, adult patients with untreated scoliosis demonstrate greater disability and lower HRQL scores compared to controls and those treated surgically. When progressive AIS has been identified, surgical intervention reliably stops curve progression and restores spinal balance, with minimal perioperative complications. Postoperatively, children surgically treated for AIS consistently demonstrate improved HRQL scores, including improved function, improved self-image and decreased pain from preoperative values. These findings support surgical treatment for progressive AIS, as non-operative measures will only allow the condition to worsen.

### "Contra" surgery

Health-related signs and symptoms of idiopathic scoliosis cannot be changed by surgery. Long-term beneficial effects of spinal fusion are not yet revealed and the long-term risks of surgery for scoliosis simply are not known. HRQL scores cannot be regarded as being valid considering the dissonance effect. Therefore the indication for spinal fusion surgery is for cosmetic reasons, only. When recognizing, that todays standard of conservative treatment can in fact lead to the same cosmetic effects as spinal fusion surgery, the latter treatment is rarely needed in patients with AIS.

## Competing interests

H–RW is currently applying for a patent relating to the Chêneau light™ off the shelf bracing system, which is demonstrated in figure 6. Additionally, he is a consultant for Koob-Scolitech. EB is a consultant for Synthes, Aesculap, and Pioneer spine implants. She furthermore declares research agreements with DePuy and Showa-Ika, and has received speakers' fees from DePuy. SB, MSW, VP, and DG did not declare any competing interests related to this paper.

## Authors' contributions

*"Pro surgery" *section: SB, VP, EB. *"Contra surgery" *section: H–RW, MSW, DG. All authors contributed equally to the general sections of this paper. All authors read and approved the final version of the manuscript.

## Consent

Written informed consent has been provided from the patients depicted in the figures, and copies of the respective consents are available for review by the editorial board.
